# Supplementary material for: Rapid increase in dichloromethane emissions from China inferred through atmospheric observations
Source: Nat Commun. 2021 Dec 14;12:7279. doi: 10.1038/s41467-021-27592-y (PMC8671471; doi:10.1038/s41467-021-27592-y)
Supplement: Supplementary file 1 — Supplementary Information [file 41467_2021_27592_MOESM1_ESM.pdf]

Supplementary information to: Rapid increase in dichloromethane emissions from China inferred through atmospheric observations

Minde An et al.

Station	Abbreviation	Longitude (°E)	Latitude (°N)	Altitude (masl)	Inlet height (magl)	Measurement periods	Sampling frequency	Location
Shangdianzi	SDZ	117.12	40.65	293	80/10	May 2010 to August 2012; December 2015-<br>September 2010-	In-situ/2h<br>Weekly flasks	North China Plain
Mt. Waliguan	WLG	100.90	36.29	3816	80	October 2010-	Weekly flasks	Qinghai-Tibet Plateau
Lin'an	LAN	119.73	30.30	138	50	September 2010-	Weekly/Daily flasks	Yangtze River Delta
Longfengshan	LFS	127.60	44.73	330	80	September 2010-	Weekly flasks	Northeast China Plain
Shangri-La	XGL	99.44	28.01	3580	50	July 2011-	Weekly flasks	Yunnan-Guizhou Plateau
Jiangjin	JGJ	106.15	29.15	262	10	March 2017-	Daily flasks	Sichuan Basin
Akedala	AKD	87.97	47.10	562	50	August 2017-	Weekly flasks	Northwest China
Jinsha	JSA	114.21	29.64	750	50	December 2018-	Weekly flasks	Central China
Xinfeng	XFG	114.17	24.08	870	50	August 2018-	Weekly flasks	Pearl River Delta

Table 1 Monitoring stations in China used in this study. 'magl' is meters above ground level, 'masl' is meters above sea level. Shangdianzi changed the sampling height from 10 m to 80 m at 19th, August, 2011. There was a three-year gap in SDZ in-situ measurement due to system malfunction. LAN started to collect daily flask samples from January 2019.

Year	Emissions in China ($Gg\ yr^{-1}$)	Emissions in the whole domain ($Gg\ yr^{-1}$)
2011	241	688
2012	242	766
2013	263	895
2014	285	967
2015	302	921
2016	317	949
2017	338	1045
2018	357	1045
2019	379	1045

Table 2 Initial values for a priori emissions in China and whole domain. The emissions in China were obtained from previous bottom-up inventory of CH_2Cl_2 emissions in China[1]. The total a priori emissions in the computational domain were taken from a previous estimate of emissions in Asia[2]. The emissions in the whole domain in 2018 and 2019 were assumed to be the same as 2017. The initial estimates of total a priori emissions for remainder of the domain were calculated as emissions in the whole domain minus emissions in China.

	Total CH_2Cl_2 (Gg)		Feedstock use (Gg)		Pharmaceutical industry (Gg)	Emissive solvent (Gg)	Coal (Tg)		Steel and iron industry (Tg)		
	CH_2Cl_2 production	CH_2Cl_2 consumption	HFC-32 production	CH_2Cl_2 consumption	CH_2Cl_2 consumption	CH_2Cl_2 consumption	Coal production	Coal consumption	Sintering production	Pig iron production	Crude steel production
2013	830	794.5	53	90.2	222.5	481.8	3974	4244	887	711	813
2014	880	844.9	52	88.9	236.6	519.5	3874	4136	901	714	822
2015	1110	983.2	84	144.4	275.3	563.5	3747	3998	870	691	804
2016	1170	1046.6	88	151.1	293	602.5	3411	3888	813	702	808
2017	1270	1124.9	151	259	315	550.9	3524	3914	839	714	871
2018	1420	1271.6	175	300.1	356	615.5	3698	3975	832	780	929
2019	1300	1113.7	186	319	311.8	482.9	3846	4019	867	808	995

Table 3 Activity levels and other relevant values to derive the production/consumption data for CH_2Cl_2 in each sector used in the bottom-up inventory. The overall CH_2Cl_2 production and consumption data were obtained from China Chlor-Alkali Industry Association (CCAIA)[3], while the consumption data is production plus import minus export. The consumption of CH_2Cl_2 used as feedstock for HFC-32 production was calculated from annual HFC-32 production (ChinaIOL[4], http://data.chinaiol.com), with a production efficiency of 95 % (following consultation with industry experts; this value is similar to that used in Feng et al.[1]). The consumption of CH_2Cl_2 used in the pharmaceutical industry is estimated to be 28 % of the overall consumption in each year[3]. All the remaining CH_2Cl_2 consumption was assumed to be emissive solvent consumption, i.e. the overall consumption minus the consumption in feedstock use and the pharmaceutical industry. The coal production and coal consumption were adapted from the China energy statistical yearbook[5]. The production values in steel and iron industry were obtained from the China steel yearbook[6]. The most up-to-date values were used in both cases.

	CH_2Cl_2 production leakage	CH_2Cl_2 feedstock use leakage	CH_2Cl_2 release from pharmaceutical industry	CH_2Cl_2 release as emissive solvent	CH_2Cl_2 release in coal production and consumption		CH_2Cl_2 release in steel and iron industry		
					production	consumption	sintering	ironmaking	steelmaking
Emission factor	0.005	0.005	0.05-0.12	1 (0.5+0.5)	10^{-9}	1.4×10^{-7}	$(0.47\text{-}2.11) \times 10^{-7}$	$(1.2\text{-}8.4) \times 10^{-9}$	$(4.8\text{-}7.2) \times 10^{-9}$

Table 4 Emission factors for CH_2Cl_2 in each sector used in the bottom-up inventory. The leakage of CH_2Cl_2 in production process or feedstock use for HFC-32 adopt the default fugitive emission factors during chemical production from IPCC (2000)[7]. There exist solvent recovery and waste treatment processes in the pharmaceutical industry, and the emission factor in this sector is estimated using mass balance and the rate of solvent waste treatment, which we assume to be 88-95%[8]. We assume the emission factor for emissive solvents is 50% in the first year and 50% in the second year. The emission factors for coal production and coal consumption were obtained from Feng et al.[1]. The emission factors in the steel and iron industry were adapted from Ding et al.[9].

Year	Emissions from China (Gg yr^{-1}) Top-down			Emissions from China (Gg yr^{-1}) Bottom-up			Global emissions (Gg yr^{-1}) Top-down		
	Mean	16th	84th	Mean	16th	84th	Mean	16th	84th
2011	231	213	245				683	541	825
2012	272	247	291				779	628	930
2013	456	412	500	506	483	531	884	705	1059
2014	452	414	483	526	508	545	910	724	1098
2015	534	477	574	572	552	592	881	697	1068
2016	466	429	497	615	593	636	933	745	1124
2017	557	522	590	612	590	632	1021	813	1227
2018	511	483	536	623	601	645	1019	810	1228
2019	628	599	658	585	564	604	1038	826	1251

Table 5 Results for global emissions and emissions from China. The "top-down" and "bottom-up" are the inversion and inventory-based estimations, respectively. The 16th and 84th are the corresponding percentile derived from the distribution of the emissions, which represents the 1 s.d. uncertainties for a Normal distribution.

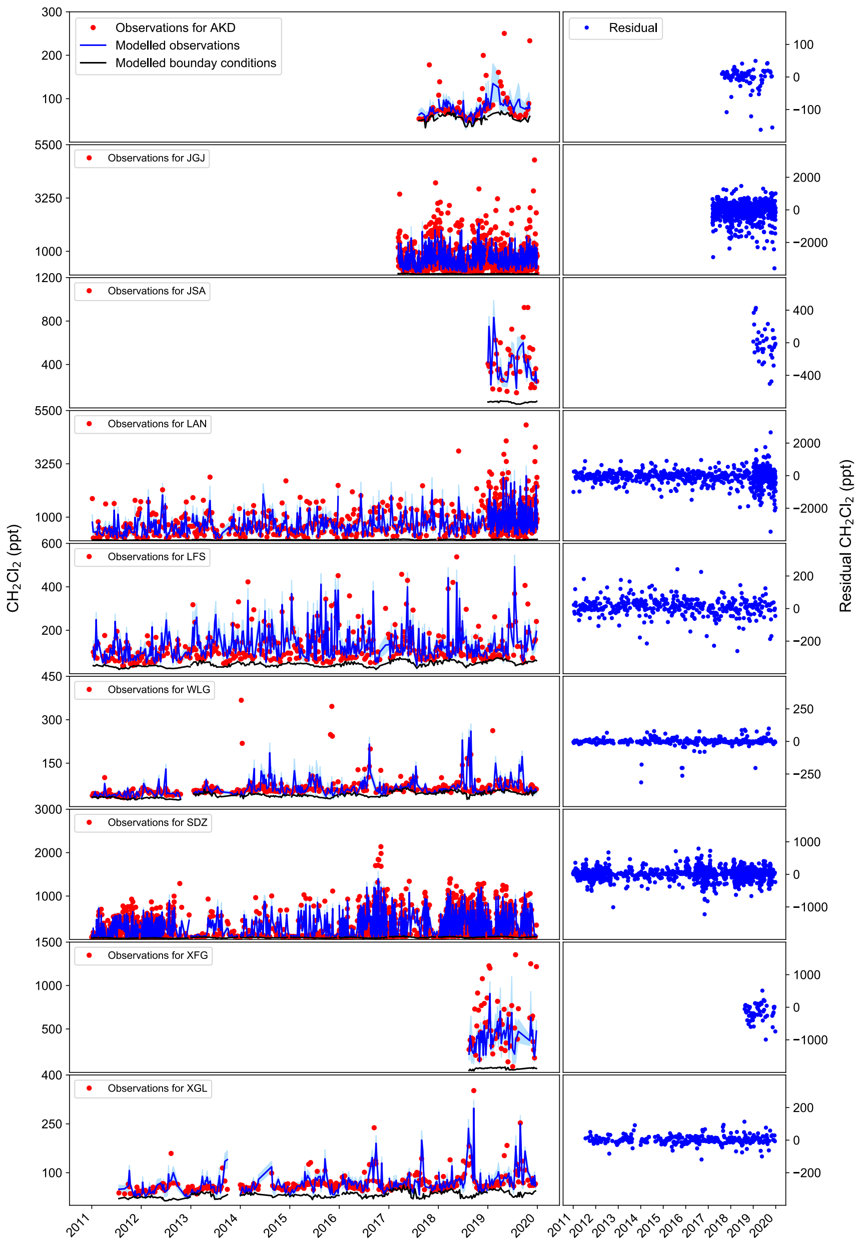

Fig. 1 Observed and simulated CH_2Cl_2 mole fractions. On the left panel, the modelled mole fractions (blue line) and their uncertainties (blue shading) derived from the inferred a posteriori emission, are compared to observation data (red dots) at each site. Modelled a posteriori mean baseline time series are also shown (black line). Observation data from the sites may indicate the magnitude of regional emission sources around each site. For some sites, such as Akedala (AKD), Shangri-la (XGL) and Waliguan (WLG), values are comparatively lower, with most measurements quite close to baseline values and pollution events up to only few hundred ppt, suggesting that there are fewer significant local sources, or that existing sources are intermittent and therefore not captured by the flasks sampling strategy, from regions to which measurements at these sites are sensitive (see Supplement Fig 6). In contrast, there are considerable enhancements above baseline for Lin'an (LAN), Jiangjin (JGJ) and Shangdianzi (SDZ), located in Yangtze River Delta area, Sichuan Basin and North China plain, respectively, indicating strong local sources in their sensitive regions. The right panel shows the residuals between observations and simulated mole fractions. This plot shows good fit for the simulation to the observations.

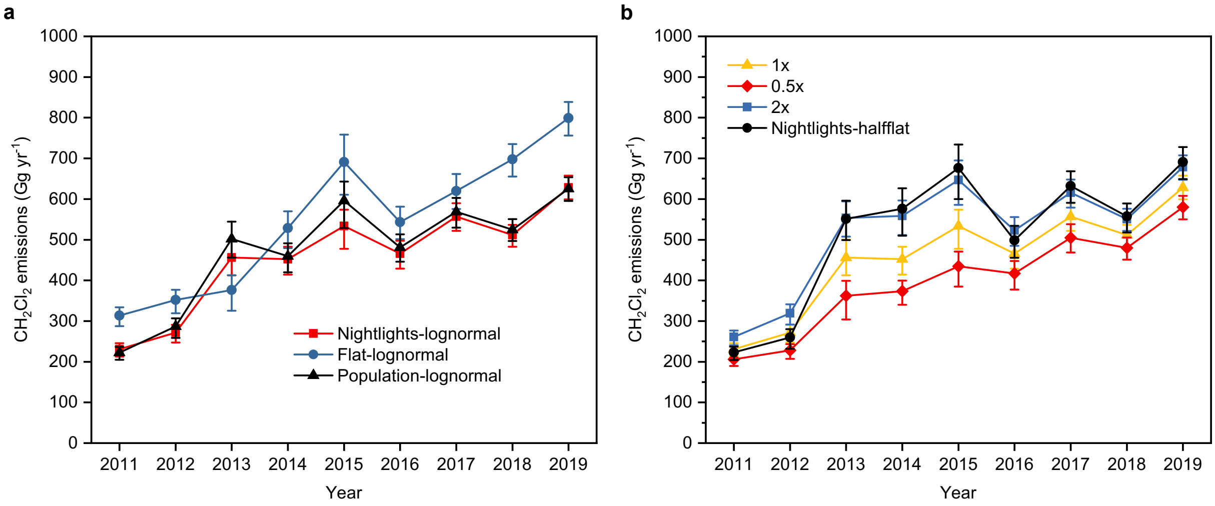

Fig. 2 Derived emissions from China using different a priori information. (a) Emissions inferred using different a priori spatial distributions. All results show similar temporal variations. The population and nightlights-based priors exhibit very similar results. The flat prior, with emissions distributed uniformly across China and outside China, has slightly higher results in later years, which may be due to the fact that our measurements have relatively poor sensitivity to some regions in China, especially Tibet in the southwest of China. A uniform prior distribution will give this area an initial value which, due to low sensitivity, may not be modified during the inversion. However, as far as we know, CH_2Cl_2 originates predominantly from anthropogenic sources, so we do not expect any signficiant emission sources from Tibet. **(b)** Emissions inferred using different initial values for a priori emissions. The uncertainty range for results of '0.5-2 time the a priori" agree well with each other, though there are large uncertainties in 2013-2015, when no in-situ measurement was conducted during this period. The black line shown in (b) is the inferred results using an uniformative a priori distribution for emissions (termed half-flat), which does not use any a priori magnitude information but forces the value to be positive. This line also shows a similar trend to the one used in the study. All the uncertainties in this plot are the 68% interval.

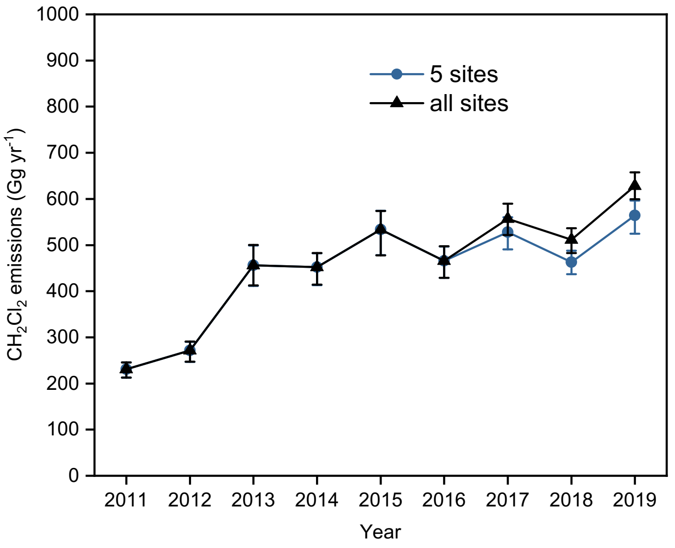

Fig. 3 Derived emissions from China using different measurement datasets. In addition to the results shown in the main text, where observations from all available measurement sites were used in the inversion (black line in this plot), inversions with observations from only 5 sites where observation persists throughout the study period (i.e. LAN, LFS, WLG, SDZ, XGL) were also conducted (blue line in this plot). Both the uncertainties are the 68% interval. The two results show similar trend.

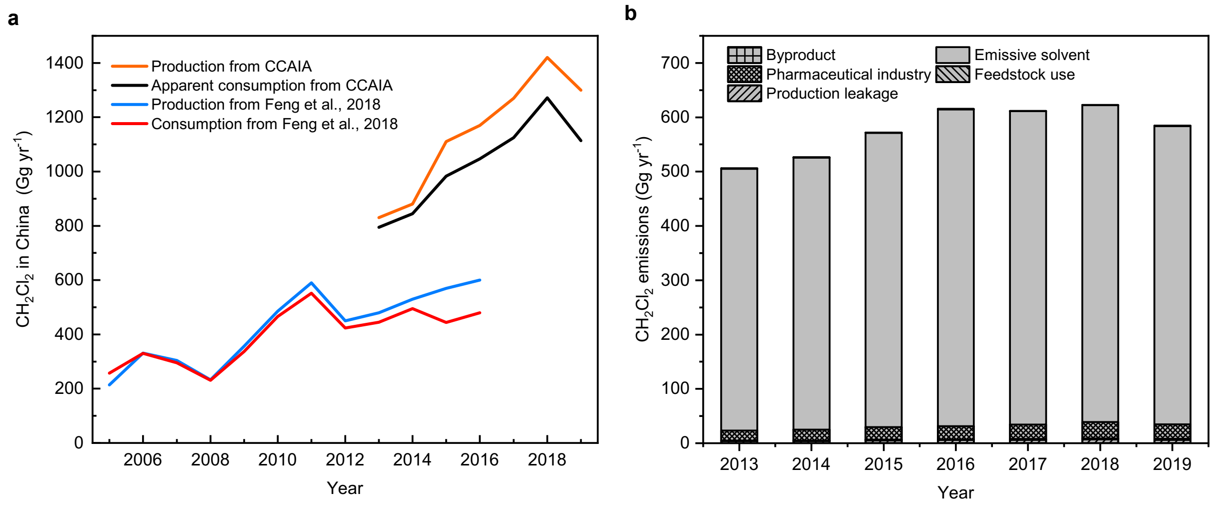

Fig. 4 Information for the bottom-up estimation. (a) Production and consumption data. Consumption (red line) and production (blue line) data used in the previously published bottom-up research for 2005-2016[1], and a new set of consumption (black line) and production (orange line) data acquired from China Chlor-Alkali Industry Association (CCAIA)[3] for 2013-2019 used in the new bottom-up estimation in this study. The consumption data from CCAIA were calculated by production + import – export and referred to as apparent consumption. **(b)** The bottom-up emission results from different sectors estimated in this study. The emissive solvent sector (including use as blowing agent for polyurethane foam manufacture) accounts for more than 90% of emissions.

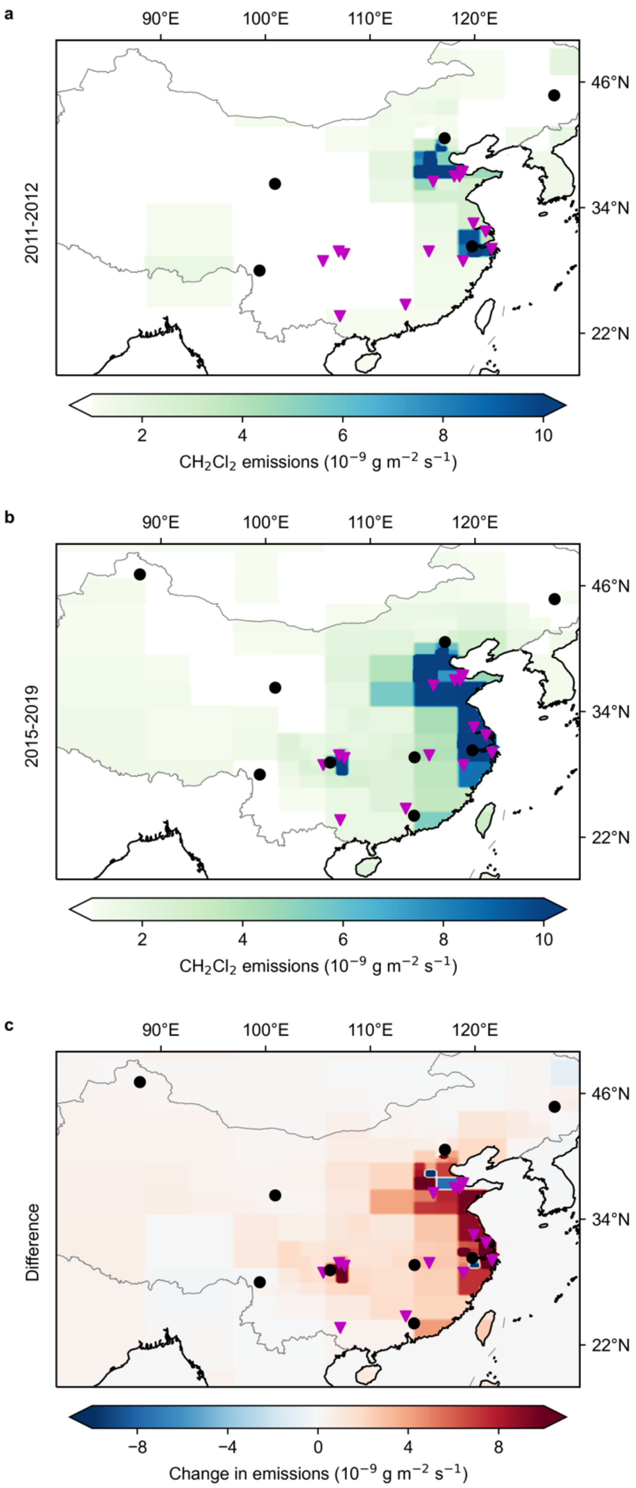

Fig. 5 Spatial distribution of CH_2Cl_2 emissions in Eastern Asia with focus on China. As seen in Fig. 3 of the main text, but using a uniform (flat) prior distribution. (a) The average mean emissions of CH_2Cl_2 in 2011-2012. **(b)** The average mean emissions of CH_2Cl_2 in 2015-2019. **(c)** The difference between (a) and (b). The black dots in the figures represent the measurement sites active in that period and the pink triangles are the known chloromethane factories in China.

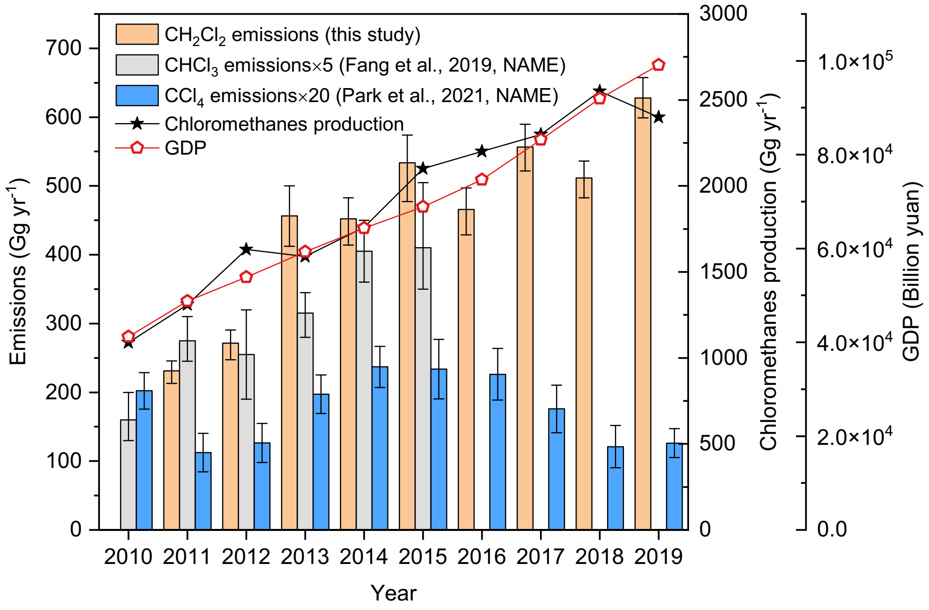

Fig. 6 Emissions of CH_2Cl_2, $CHCl_3$[10] and CCl_4[11] in China. From previous papers, if emissions were estimated with multiple methods, the results using NAME are adopted for comparison. Emissions of $CHCl_3$ and CCl_4 are for eastern China only. Emissions of $CHCl_3$ have been multiplied by five, and CCl_4 emissions by twenty, for ease of comparison. Total chloromethanes production (combination of CH_2Cl_2 and $CHCl_3$) in China is also shown. The GDP represents for Gross Domestic Product of China, obtained from the yearbook[12].

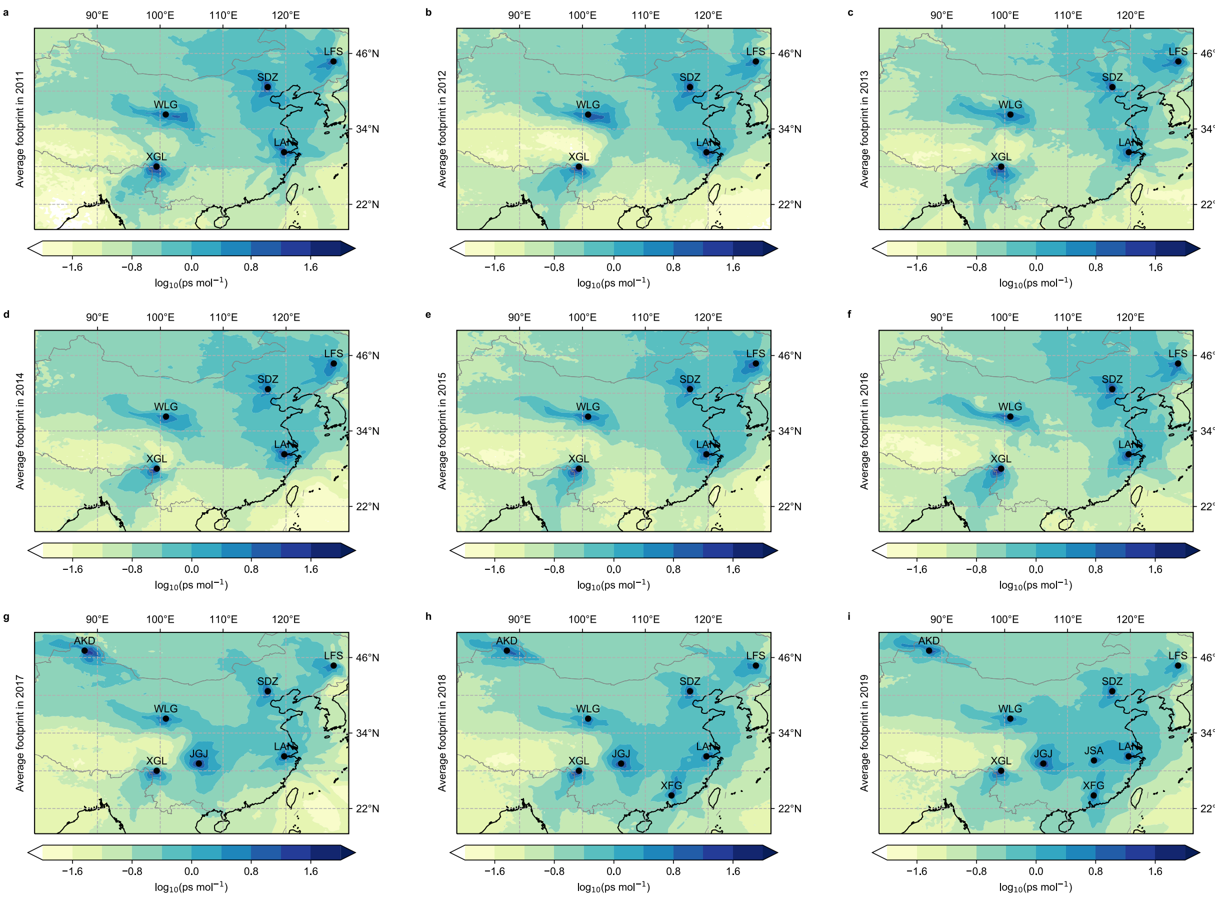

Fig. 7 Average sensitivity of the observations to emissions of CH_2Cl_2 in each year. Each plot shows the mean result of averaged footprints from all sites in that year when measurements were conducted at that site. For most of the regions, including the eastern part of China where substantial emissions are thought to occur, the mean sensitivity did not change substantially throughout the period.

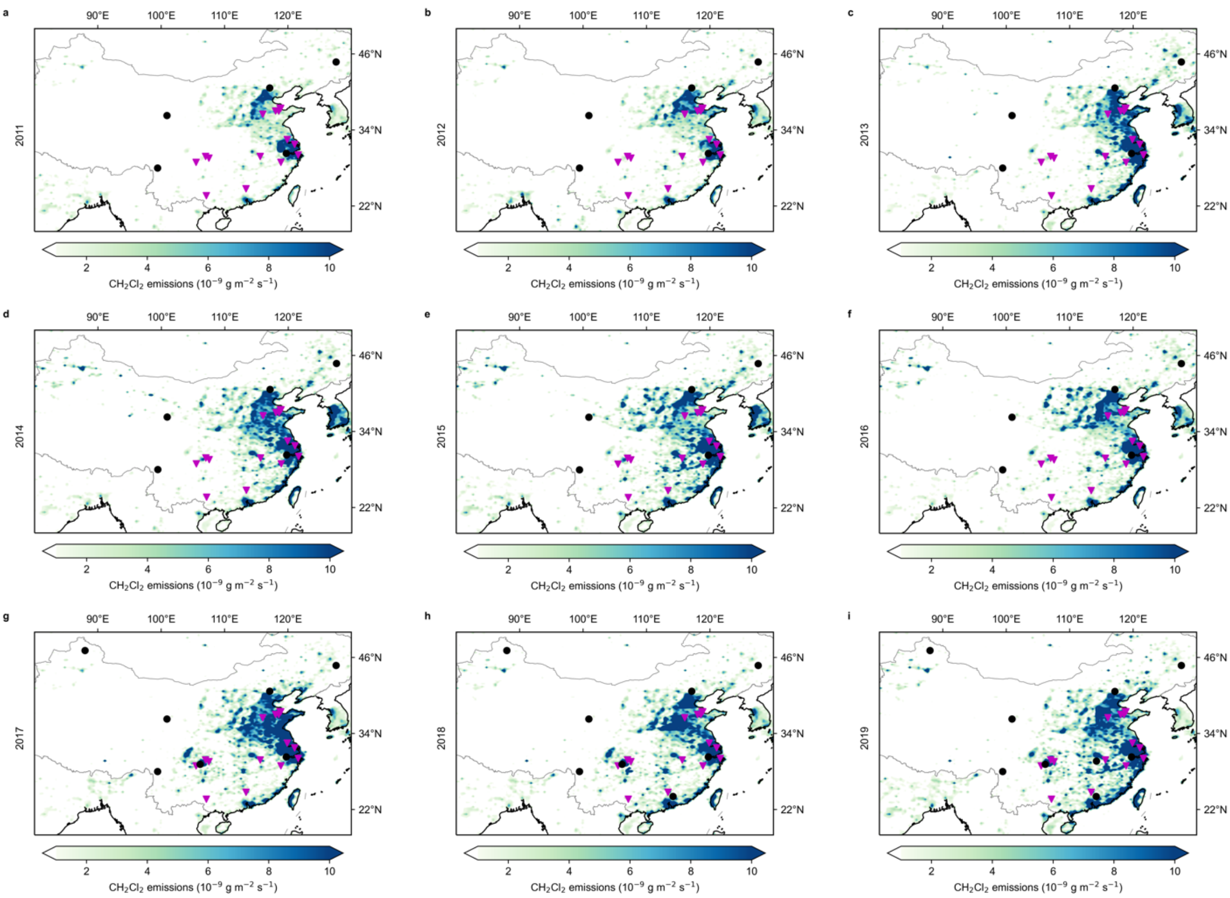

Fig. 8 Spatial distribution of CH_2Cl_2 emissions in Eastern Asia with focus on China during 2011-2019. The black dots in the figures represent the measurement sites active in that year; the pink triangles are the known chloromethanes factories in China.

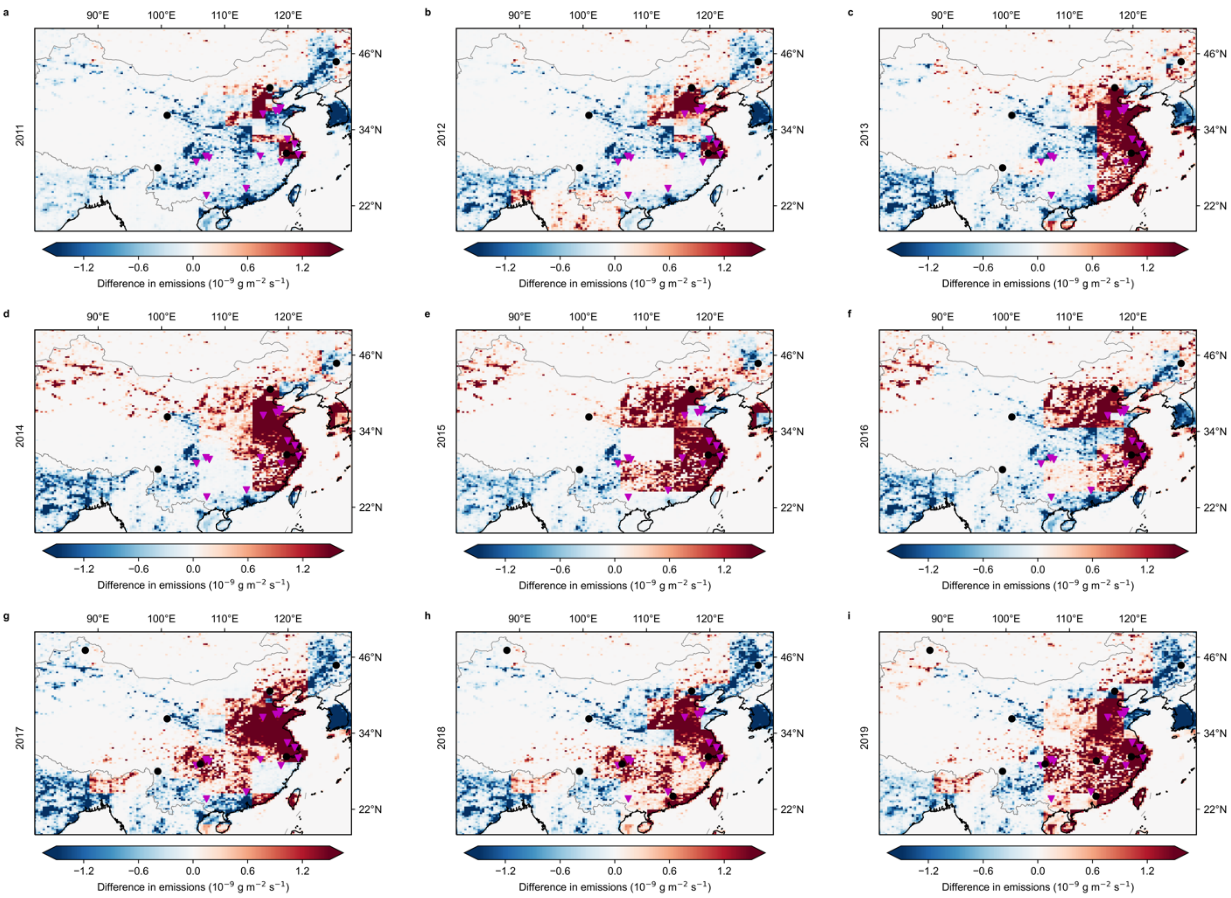

Fig. 9 Spatial distribution of the difference between the final results and the a priori emissions for CH_2Cl_2 during 2011-2019. These plots show the difference between the a posteriori mean emission distribution after the inversion and the a priori emission distribution. The black dots in the figures represent the measurement sites active in that year. The pink triangles are the known chloromethanes factories in China. We can see that before the rapid increase (before 2013), only part of Yangtze River Delta and North China plain have greater emissions, relative to the a priori emissions, while after 2013 a wider area of the Yangtze River Delta and North China plain are significantly larger than the a priori emissions.

References

1. Feng, Y., Bie, P., Wang, Z., Wang, L. & Zhang, J. Bottom-up anthropogenic dichloromethane emission estimates from China for the period 2005–2016 and predictions of future emissions. *Atmos. Environ.* **186**, 241–247 (2018).
2. Claxton, T. *et al.* A synthesis inversion to constrain global emissions of two very short lived chlorocarbons: dichloromethane, and perchloroethylene. *J. Geophys. Res. Atmos.* **125**, e2019JD031818 (2020).
3. China Chlor-Alkali Industry Association (CCAIA). Report on chloromethanes industry in China (in Chinese). http://www.ccaon.com/jwlhw.asp (2021).
4. ChinaIOL. China's HFC-32 production (in Chinese). http://data.chinaiol.com (2021).
5. Department of Energy Statistics, National Bureau of Statistics. *China Energy Statistical Yearbook (in Chinese)*. (China Statistics Press, Beijing, China, 2020).
6. China Iron and Steel Industry Association. *China Steel Yearbook (in Chinese)*. (The editorial board of China steel yearbook, Beijing, China, 2018-2020).
7. Intergovernmental Panel on Climate Change (IPCC). *Good Practice Guidance and Uncertainty Management in National Greenhouse Gas Inventories*. (2000).
8. Song, Y. Study on emission standard of volatile organic compounds and malodorous gases from penicillin industry (in Chinese). (Hebei University of Science and Technology, 2014).
9. Ding, X. *et al.* Gaseous and particulate chlorine emissions from typical iron and steel industry in China. *J. Geophys. Res. Atmos.* **125**, e2020JD032729 (2020).
10. Fang, X. *et al.* Rapid increase in ozone-depleting chloroform emissions from China. *Nat. Geosci.* **12**, 89–93 (2019).
11. Park, S. *et al.* A decline in emissions of CFC-11 and related chemicals from eastern China. *Nature* **590**, 433–437 (2021).
12. National Bureau of Statistics of China. *China Statistical Yearbook*. (China Statistics Press, Beijing, China, 2020).
