# Supplementary material for: Rapid increase in dichloromethane emissions from China inferred through atmospheric observations
Source: Nat Commun. 2021 Dec 14;12:7279. doi: 10.1038/s41467-021-27592-y (PMC8671471; doi:10.1038/s41467-021-27592-y)
Supplement: Supplementary file 3 — Description of Additional Supplementary Files [file 41467_2021_27592_MOESM3_ESM.docx]

**Description of Additional Supplementary Files

Supplementary Data 1.** The file contains the measured mole fractions used in the regional emission inversion framework. All times in the file are UTC. The mole fraction units are parts per trillion (ppt).

**Supplementary Data 2.** The file contains the provincial results (which includes the provinces on the Chinese mainland excluding Hong Kong and Macao) for CH_2_Cl_2_ emissions in China. “CHINA_prior” is the a priori magnitude for emissions in China. Columns named with a "mean" suffix are the posterior mean value from the Markov chain in each province/China. Columns named with a "16%" or "84%" suffix are the bounds of the 68% uncertainty interval for the posterior value in each province/China. Columns named with a "2.5%" or "97.5%" suffix are the 95% uncertainty interval for the posterior value in each province/China. The units are Gg yr^-1^.
